# IL-17 Induced Autophagy Regulates Mitochondrial Dysfunction and Fibrosis in Severe Asthmatic Bronchial Fibroblasts

**DOI:** 10.3389/fimmu.2020.01002

**Published:** 2020-05-21

**Authors:** Rakhee K. Ramakrishnan, Khuloud Bajbouj, Saba Al Heialy, Bassam Mahboub, Abdul Wahid Ansari, Ibrahim Y. Hachim, Surendra Rawat, Laila Salameh, Mahmood Y. Hachim, Ronald Olivenstein, Rabih Halwani, Rifat Hamoudi, Qutayba Hamid

**Affiliations:** ^1^Sharjah Institute for Medical Research – College of Medicine, University of Sharjah, Sharjah, United Arab Emirates; ^2^College of Medicine, Mohammed Bin Rashid University, Dubai, United Arab Emirates; ^3^Meakins-Christie Laboratories, McGill University, Montreal, QC, Canada; ^4^Rashid Hospital, Dubai Health Authority, Dubai, United Arab Emirates

**Keywords:** severe asthma, bronchial fibroblasts, mitochondria, autophagy, IL-17, fibrosis, mitochondrial dysfunction

## Abstract

The accumulation of fibroblasts, their synthesis of extracellular matrix (ECM) proteins and their innate resistance to apoptosis are characteristics of subepithelial fibrosis observed in severe asthma. Interleukin-17 (IL-17) is an important regulator of airway remodeling in asthma. However, the contribution of IL-17 to the pro-fibrotic phenotype of bronchial fibroblasts is not well-characterized. In this study, we investigated whether IL-17 induced autophagy regulates mitochondrial and pro-fibrotic function in bronchial fibroblasts. The primary cultured bronchial fibroblasts isolated from non-asthmatic (NHBF) and severe asthmatic (DHBF) subjects were treated with IL-17 in order to ascertain its effect on mitochondrial function, mitochondrial quality control, and apoptosis using immunoblotting and flow cytometric analyses. At baseline, DHBF exhibited higher levels of mitophagy and mitochondrial biogenesis compared to NHBF. Immunohistochemical evaluation of bronchial biopsies showed intense PINK1 immunoreactivity in severe asthma than in control. IL-17 intensified the mitochondrial dysfunction and impaired the mitochondrial quality control machinery in NHBF and DHBF. Moreover, IL-17 augmented a pro-fibrotic and anti-apoptotic response in both group of fibroblasts. Inhibition of autophagy using bafilomycin-A1 reduced PINK1 expression in NHBF and restored the IL-17 mediated changes in PINK1 to their basal levels in DHBF. Bafilomycin-A1 also reversed the IL-17 associated fibrotic response in these fibroblasts, suggesting a role for IL-17 induced autophagy in the induction of fibrosis in bronchial fibroblasts. Taken together, our findings suggest that IL-17 induced autophagy promotes mitochondrial dysfunction and fibrosis in bronchial fibroblasts from both non-asthmatic and severe asthmatic subjects. Our study provides insights into the therapeutic potential of targeting autophagy in ameliorating fibrosis, particularly in severe asthmatic individuals.

## Introduction

Fibroblasts, the effector cells of fibrosis, exhibit a bio-synthetic, contractile, adhesive, and pro-inflammatory phenotype for effective wound healing. As opposed to their self-limited and tightly regulated repair in response to tissue injury, under pathological conditions, persistent fibroblast activation paves way to extracellular matrix (ECM) accumulation, and remodeling along with their differentiation into apoptosis-resistant myofibroblasts. Chronic lung diseases, such as asthma, chronic obstructive pulmonary disease (COPD), and idiopathic pulmonary fibrosis (IPF), exhibit phenotypically different fibroblasts that are responsible for the loss of the typical airway architecture and impair airway function (1).

Increased airway fibroblast population, increased collagen deposition, airway smooth muscle hyperplasia, and hypertrophy are characteristic airway structural changes that selectively differentiate severe persistent asthma from milder forms of the disease (2). The fibroblast numbers and collagen deposition also negatively correlate with the extent of airflow limitation in patients with asthma (2). In addition to the central airways, higher myofibroblast numbers were reported in the alveolar and lung parenchyma of asthmatics (3, 4). Nonetheless, fibroblasts and their role in asthma pathogenesis have been relatively undervalued and understudied.

Th17 cells and their canonical cytokines, IL-17A and IL-17F, are key players in the pathogenesis of asthma and are closely associated with the more severe phenotypes (5). Airway tissues from patients with severe asthma demonstrated increased expression of Th17-associated cytokines, IL-17A, and IL-17F (6), together with increased expression of IL-8 and excess neutrophilia (7). IL-17 being a key mediator of neutrophilic inflammation, upregulated IL-17 expression can be considered a characteristic hallmark of severe asthma, known for exhibiting a Th2-low, and neutrophilic phenotype (8). IL-17 induced the secretion of pro-fibrotic cytokines and pro-inflammatory mediators, including IL-6, IL-11, IL-8, and GROα/CXCL1, by bronchial fibroblasts exerting their importance in regulating fibrotic and inflammatory responses in the airways (9). Furthermore, bronchial fibroblasts when co-cultured with CD4+ T cells promoted a Th17 profile in asthma (10). It was also reported that anti-IL-17 therapy in a murine asthma model exacerbated with lipopolysaccharide led to decreased oxidative stress and ECM remodeling (11) further implicating IL-17 in airway remodeling in asthma.

Increasing evidence suggests that mitochondrial dysfunction is key to the pathogenesis of asthma (12–14). Exposure to environmental oxidants such as tobacco smoke, diesel exhaust particles, and ozone caused an increase in cellular reactive oxygen species (ROS) levels which subsequently induced mitochondrial dysfunction in the lung (15). An experimental allergic model of asthma demonstrated mitochondrial dysfunction in lung mitochondria and associated mitochondrial structural changes in bronchial epithelium (13). Oxidative damage-induced mitochondrial dysfunction further exacerbated allergic airway inflammation (14). That said, this may be a strong indication that beyond its canonical function of ATP production, the non-canonical roles of mitochondria can also influence airway structure and function.

IL-17 mediated mitochondrial dysfunction has been studied in disease models, including rheumatoid arthritis (RA) (16) and vitiligo (17). However, there exists a gap in knowledge regarding the role of IL-17 in mitochondrial dysfunction in asthma, and more importantly in whether this affects airway remodeling in severe asthma. In the present study, we investigated the putative link between autophagy and IL-17A induced mitochondrial dysfunction and fibrosis in non-asthmatic and severe asthmatic (S-As) fibroblasts. IL-17A will henceforth be referred to as IL-17 in the rest of this study. Using bronchial fibroblasts isolated from S-As and non-asthmatic subjects, we show increased autophagy, mitochondrial dysfunction, and fibrotic gene expression in S-As fibroblasts which was exacerbated upon stimulation with IL-17, thereby contributing to the pathobiology of subepithelial fibrosis in severe asthma.

## Materials and Methods

### Cell Culture

The primary bronchial fibroblasts were isolated from endobronchial tissue biopsies obtained from non-smoking patients with severe asthma or non-smoking healthy volunteers. The healthy and severe asthmatic subjects were age-matched to exclude the confounding effects due to age. The mean age of the healthy and severe asthmatic subjects was 43.7 ± 12.5 and 43.4 ± 8.3 years, respectively. These fibroblasts were obtained from the Quebec Respiratory Health Research Network (McGill University Health Centre/Meakins-Christie Laboratories Tissue Bank, Montreal, Canada), as described previously (18). The cells were cultured in Dulbecco's modified Eagle's medium (DMEM) supplemented with 10% fetal bovine serum (FBS), 2 mM L-glutamine, 100 units/ml of penicillin, and 100 ng/ml streptomycin in 75-cm^2^ flasks. The cells were maintained at 37°C in 5% CO_2_ with medium change performed every 2–3 days. The fibroblasts were passaged a maximum of eight times and experiments were conducted using fibroblasts at matched passages. All cell culture reagents were purchased from Sigma-Aldrich.

The cells were seeded in 6- or 12-well-plates for experiments and at 50% confluency, they were serum-starved in 1% FBS-supplemented DMEM for 24 hours (h). The cells were then stimulated with 25 ng/ml recombinant human IL-17A (Sigma) for 48 h (for mRNA) or 96 h (for protein). Autophagy inhibition was achieved by pre-treating cells with 10 nM bafilomycin-A1 (Santa Cruz) for 4 h prior to stimulation with IL-17. Co-treatment with 10 μM carbonyl cyanide-4-(trifluoromethoxy) phenylhydrazone (FCCP) (Tocris) for 2 h was used as a positive control to induce mitochondrial dysfunction.

### Immuno/Western Blotting

The cells were lysed using 10X RIPA Buffer (abcam) after supplementation with 1x Protease Inhibitor Cocktail (Sigma-Aldrich) and 1 mM phenylmethylsulfonyl fluoride (Sigma-Aldrich). Total protein concentrations were determined using Protein Assay Kit II (Bio-Rad). Twenty micrograms total proteins were separated using either 12.5% gels or 4–20% Mini-PROTEAN TGX Precast Protein Gels (Bio-rad). The proteins were transferred onto a nitrocellulose membrane (Bio-rad), blocked in skimmed milk for 1 h at room temperature, incubated overnight at 4°C with antibodies specific to LC3B (abcam), Mitophagy Antibody Sampler Kit (Cell Signaling Technology), LAMP2A (abcam), SIRT1 (Cell Signaling Technology), PGC1α (Novus Biologicals), and Survivin (abcam). β-actin (Sigma-Aldrich) or GAPDH (Cell Signaling Technology) were used as loading controls. The blots were developed using the Clarity Western ECL Substrate (Bio-Rad) in the ChemiDoc Touch Gel Imaging System (Bio-Rad). Image Lab software (Bio-Rad) was used to detect and quantify the protein bands.

### Mitochondrial Assays

The mitochondrial mass and mitochondrial membrane potential-associated apoptosis were measured using MitoTracker Green (Invitrogen) and Mitochondrial Membrane Potential Apoptosis Kit, with Mitotracker Red & Annexin V Alexa Fluor 488 (Invitrogen), respectively. The cells were stained with 50 nM MitoTracker Green or 50 nM MitoTracker Red while protected from light for 30 min at 37°C in an atmosphere of 5% CO_2_. The MitoTracker Red stained cells were thereafter washed and labeled with Annexin V-AF488 for 15 min at room temperature in the dark. The cells were then analyzed using the BD FACSAria III flow cytometer and the acquired data analyzed using FlowJo v10 software.

### Immunohistochemistry

Paraffin slides of bronchial biopsy tissues obtained by fiberoptic bronchoscopy from non-asthmatic control subjects archived at the Biobank of the Quebec Respiratory Health Research Network Canada with MUHC REB number BMB-02-039-t (19), were obtained. Severe asthmatic subjects, who fulfilled the American Thoracic Society (ATS) criteria and were taking treatments based on the Global Initiative for Asthma (GINA), were recruited by the treating physician and nurse who obtained written informed consent, from the Severe Asthma Clinic in the Pulmonary Medicine department at Rashid Hospital, Dubai, UAE. The endobronchial biopsies were performed in accordance with a study protocol approved by the Dubai Scientific Research Ethics Committee with approval number DSREC-11/2017_04. The biopsies were collected and embedded as previously described (20).

Immunohistochemical staining of formalin-fixed paraffin-embedded (FFPE) biopsy samples was performed to determine the protein levels and distribution of PINK1 as previously described (20). Briefly, 3 μm thick sections were cut from the paraffin blocks and routine deparaffinization and rehydration steps performed. Heat-activated antigen retrieval was carried out using sodium citrate buffer at pH 6.0 and developed using HRP/DAB (ABC) Detection IHC kit (abcam), according to manufacturer recommendations. The slides were immunostained with rabbit anti-PINK1 (1:350 dilution; Novus Biologicals) antibody. The primary antibody was omitted to serve as technical negative control and appropriate positive control tissue was used. Nuclei were counterstained blue with hematoxylin (Thermo Scientific Shandon).

### Quantitative Polymerase Chain Reaction (qPCR)

The total RNA was extracted using the Trizol (Invitrogen) method according to manufacturer instructions. RNA concentrations were measured using Nanodrop spectrophotometer (Thermo Scientific). Reverse transcription was performed using High-Capacity cDNA Reverse Transcription Kit (Applied Biosystems) in the Veriti Thermal Cycler (Applied Biosystems). cDNA amplification was carried out using 5x Hot FirePol EvaGreen qRT-PCR SuperMix (Solis Biodyne) in QuantStudio 3 Real-Time PCR System (Applied Biosystems). The primers are listed in Table 1. Gene expression was analyzed using the Comparative Ct (ΔΔCt) method after normalization to the housekeeping gene 18 s rRNA. All results were expressed as fold change relative to NHBF for baseline measurements or the untreated controls for IL-17 treatment.

**Table 1:** List of primer sequences used in qPCR.

**Genes**	**Forward primer sequence**	**Reverse primer sequence**
	**(5**′**-3**′**)**	**(5**′**-3**′**)**
IL-6	GAAAGCAGCAAAGAGGCAC	GCACAGCTCTGGCTTGTTCC
BECN1	ATGCAGGTGAGCTTCGTGTG	CTGGGCTGTGGTAAGTAATGGA
ATG5	GACCAGTTTTGGGCCATCAATC	GTGCAACTGTCCATCTGCAGC
LC3B	GAACGGACACAGCATGGTCAGC	ACGTCTCCTGGGAGGCATAG
SQSTM1	TTGTACCCACATCTCCCGCCA	TACTGGATGGTGTCCAGAGCCG
LAMP2	AACTTCAACAGTGGCACCCACC	AGTGATGTTCAGCTGCAGCCCC
PINK1	CCTGCGCCAGTACCTTTGTGT	TGGGTCCAGCTCCACAAGGATG
PRKN	CTCCAGCCATGGTTTCCCAGTG	CCAGGTCACAATTCTGCACAGTC
COL1A1	GATTGACCCCAACCAAGGCTG	GCCGAACCAGACATGCCTC
COL3A1	GATCAGGCCAGTGGAAATG	GTGTGTTTCGTGCAACCATC
COL5A1	GTCGATCCTAACCAAGGATGC	GAACCAGGAGCCCGGGTTTTC
FN1	CTGGGAACACTTACCGAGTGGG	CCACCAGTCTCATGTGGTCTCC
ACTA2	CTTCGTGTTGCCCCTGAAGAG	GCATAGAGAGACAGCACCGC
18s	TGACTCAACACGGGAAACC	TCGCTCCACCAACTAAGAAC

### Statistical Analysis

All data are presented as mean ± standard error of the mean (SEM) of 2–4 independent experiments. Data analyses were performed using Mann Whitney test while comparing NHBF and DHBF, one-way ANOVA followed by Tukey's multiple comparison tests or unpaired *t*-test with multiple comparisons using the Holm-Sidak method for statistical analysis of the data using GraphPad Prism 6 software (GraphPad, San Diego, CA, USA). A *p* < 0.05 was considered statistically significant.

## Results

### Enhancement of Mitochondrial Quality Control in S-As Fibroblasts

**Figure 1:**
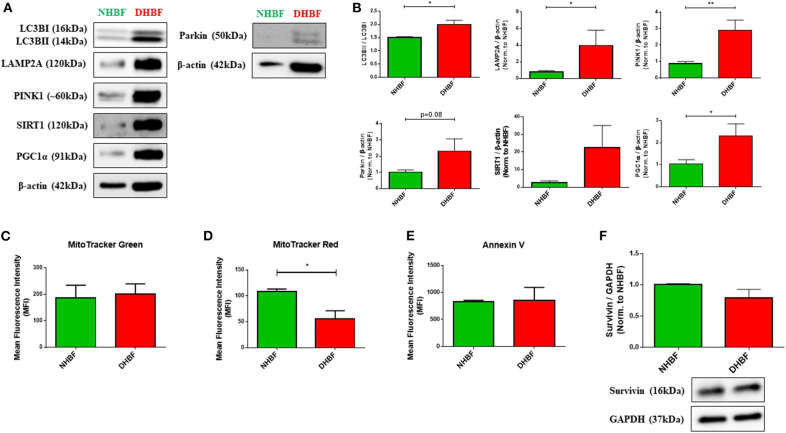
Enhancement of mitochondrial quality control in severe asthmatic (S-As) fibroblasts. In order to measure the basal expression levels, non-asthmatic fibroblasts (NHBF), and S-As fibroblasts (DHBF) were serum-starved for 24 h and thereafter, cultured in DMEM complete medium for 24 h. The fibroblasts were then subjected to Western Blot or flow cytometric analysis. β-actin or GAPDH was used as loading control as indicated. **(A)** Representative immunoblots and **(B)** densitometric analysis of autophagy markers, with LC3B lipidation represented as the ratio of LC3BII to LC3BI, and LAMP2A expression, mitophagy markers, PINK1 and Parkin, and mitochondrial biogenesis markers, SIRT1 and PGC1α. Data representative of three independent experiments. **(C)** Bar charts indicating NHBF (green) and DHBF (red) fluorescence when stained with MitoTracker Green, **(D)** MitoTracker Red and **(E)** Annexin V followed by flow cytometric analysis. 10,000 events were analyzed in each flow cytometry experiment. Data representative of three independent experiments. **(F)** Densitometric analysis and representative immunoblots depicting expression of anti-apoptotic protein, Survivin, in NHBF and DHBF. Data presented as mean ± SEM relative to NHBF (where indicated). Statistical significance assessed by Mann Whitney test. *p < 0.05, **p < 0.01.

Being highly dynamic organelles, mitochondria are constantly under the surveillance of mitochondrial quality control (QC) mechanisms of mitophagy, and mitochondrial biogenesis to identify and resolve mitochondrial defects. To investigate the state of mitochondrial dysfunction in S-As fibroblasts, the mitochondrial QC mechanisms were examined in S-As fibroblasts (DHBF) and non-asthmatic fibroblasts (NHBF) isolated from endobronchial biopsy tissues. Mitophagy is triggered by the accumulation of PTEN-induced putative kinase protein 1 (PINK1) on the outer mitochondrial membrane as a result of mitochondrial depolarization. PINK1 then recruits E3 ubiquitin ligase Parkin which ubiquitinates mitochondrial surface proteins tagging them for autophagy-dependent lysosomal clearance. Since mitophagy is dependent on the autophagy machinery, we first examined the protein expression of autophagy marker, microtubule-associated protein 1 light chain 3 beta (LC3B), and lysosome-associated membrane protein 2A (LAMP2A), which is essential for lysosomal fusion with autophagic vacuoles (21). In comparison to NHBF, increased accumulation of LC3B, increased LC3B lipidation (conversion of LC3BI to LC3BII) (*p* = 0.03), and upregulated expression of LAMP2A (*p* = 0.02) were detected in DHBF (Figures 1A,B).

Western blot analysis also showed increased expression of mitophagy-specific proteins, PINK1 (*p* = 0.004) and Parkin (*p* = 0.08) in DHBF compared to NHBF (Figures 1A,B), which indicated increased levels of mitophagy in S-As fibroblasts. Higher expression of mitochondrial biogenesis markers, sirtuin 1 (SIRT1), and proliferator-activated receptor gamma co-activator 1-alpha (PGC-1α) (*p* = 0.02), was also detected in DHBF than in NHBF (Figures 1A,B). This activation of mitochondrial QC mechanisms in S-As fibroblasts is indicative of intrinsic mitochondrial damage.

MitoTracker dyes are a useful tool in assessing mitophagy (22). Therefore, we next evaluated the total mitochondrial mass using MitoTracker Green, a fluorescent dye that binds mitochondria independent of mitochondrial membrane potential (ΔΨm), and the mitochondrial membrane potential was determined using the MitoTracker Red fluorescent probe. MitoTracker Green staining showed similar mitochondrial mass in NHBF and DHBF (Figure 1C), which supported the increased turnover of damaged mitochondria in S-As fibroblasts through enhanced mitophagy and biogenesis. Furthermore, MitoTracker Red staining also showed approximately 48% reduction in ΔΨm in DHBF (*p* = 0.03) when compared to NHBF (Figure 1D), confirming the presence of mitochondrial abnormalities in S-As fibroblasts.

Studies have shown that mitochondrial damage and autophagy are closely related to cell death (23, 24). We, therefore, studied mitochondrial damage-mediated cell apoptosis by using Annexin V staining to detect apoptotic cells. Flow cytometric analysis showed comparable Annexin V staining in NHBF and DHBF (Figure 1E). This was also corroborated in the western blot analysis of anti-apoptotic protein survivin, which showed similar expression levels in NHBF and DHBF (Figure 1F). Taken together, these findings may imply that mitochondrial damage in S-As fibroblasts is associated with efficient recycling of mitochondria through mitophagy and biogenesis resulting in fibroblast resistance to apoptosis.

### Increased PINK1 Expression in Severe Asthmatic Bronchial Biopsy Tissues

**Figure 2:**
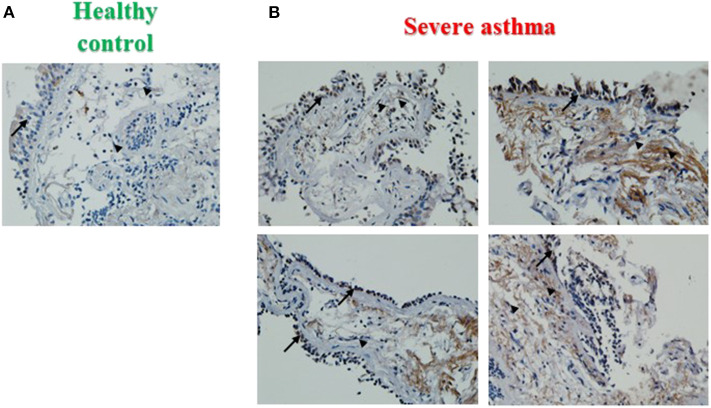
Increased PINK1 expression in severe asthmatic bronchial biopsy tissues. Representative images taken at 40X magnification showing PINK1 immunostaining developed with 3,3′-diaminobenzidine diaminobenzidine (brown). Nuclei were counterstained with hematoxylin (blue). Representative bronchial biopsy sections from **(A)** healthy control showing weak, **(B)** severe asthmatic showing moderate to strong PINK1 protein expression. Arrows refer to bronchial epithelium. Arrowheads refer to fibroblasts.

PINK1 is fundamental to mitochondrial homeostasis and serves as a sensor of mitochondrial damage (25). For further validation of the role of mitochondrial dysfunction in asthma progression, we next performed immunohistochemical staining of PINK1 on bronchial biopsies obtained from a cohort of six patients with severe asthma compared to 2 biopsies obtained from healthy control subjects. Our results showed that PINK1 protein levels were high (moderate to strong) in the bronchial epithelium as well as in fibroblasts of four out of the six (66.67%) severe asthmatic patients' samples (Figure 2B) compared to the healthy control group that showed low expression levels (negative or weak stain) (Figure 2A). PINK1 immunostaining across the 8 biopsies is summarized in Table 2. Thus, PINK1 serves a protective role in S-As fibroblasts by defending cells from damage-mediated mitochondrial dysfunction and cellular apoptosis.

**Table 2:** PINK1 immunohistochemistry staining.

	**Negative - weak**	**Moderate - strong**
Healthy control	2 (100%)	0 (0%)
Severe asthma	2 (33.34%)	4 (66.67%)

### IL-17 Increases Mitochondrial Dysfunction in Bronchial Fibroblasts

**Figure 3:**
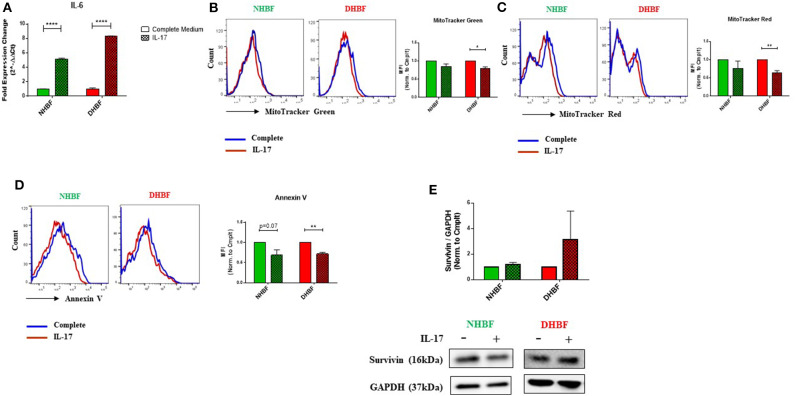
IL-17 increases mitochondrial dysfunction in bronchial fibroblasts. NHBF and DHBF were serum-starved for 24 h and thereafter, cultured in DMEM complete medium without or with IL-17 (25 ng/ml) for up to 96 h. **(A)** mRNA expression of IL-6 in NHBF and DHBF upon IL-17 stimulation was analyzed by qPCR and expressed as fold expression change relative to the respective untreated control post normalization to housekeeping gene 18 s rRNA. Data representative of *n* = 3. **(B)** Representative histograms and bar charts indicating fluorescence in mean fluorescence intensity (MFI) in cells stained with MitoTracker Green, **(C)** MitoTracker Red, and **(D)** Annexin V followed by flow cytometric analysis. 10,000 events were analyzed in each flow cytometry experiment. Data representative of three independent experiments. **(E)** Densitometric analysis and representative immunoblots depicting expression of anti-apoptotic protein, Survivin, in NHBF (left panel) and DHBF (right panel) upon IL-17 stimulation. GAPDH was used as loading control. Data presented as mean ± SEM after normalization to the respective untreated control (Complete Medium). Statistical significance assessed by unpaired *t*-test with multiple comparisons using the Holm-Sidak method. *p < 0.05, **p < 0.01, ****p < 0.0001.

Since IL-17 is strongly implicated in the pathogenesis of severe asthma (5), we investigated the effects of IL-17 on mitochondrial mass and function in both non-asthmatic NHBF and severe asthmatic DHBF. Stimulation of bronchial fibroblasts with IL-17 at a concentration of 25 ng/ml was shown to activate inflammatory and remodeling processes (26). In view of the fact that bronchial airway tissue is subjected to chronic exposure to IL-17 in patients with severe asthma (6), NHBF and DHBF were incubated with 25 ng/ml of IL-17 for a duration of up to 96 h to study the long-term effect of prolonged exposure to IL-17. A 48-h exposure to IL-17 significantly increased the mRNA levels of IL-6 in both NHBF and DHBF (Figure 3A), which indicated 25 ng/ml of IL-17 to be an effective dose to study the pathology associated with IL-17 in NHBF and DHBF.

IL-17 mediated mitochondrial dysfunction is usually associated with mitochondrial depolarization (16). We, therefore, examined whether IL-17 caused changes in mitochondrial quantity and quality in NHBF and DHBF. MitoTracker Green and mitochondrial membrane potential-dependent MitoTracker Red staining showed an inclination towards a decline in NHBF upon stimulation with IL-17 (Figures 3B,C), where their normalized fluorescence decreased from 1 to 0.85 and 0.77, respectively. IL-17, however, significantly attenuated mitochondrial mass and ΔΨm in DHBF when compared to untreated cells (Figures 3B,C). In DHBF, IL-17 caused a drop in normalized MitoTracker Green and MitoTracker Red fluorescence from 1 to 0.79 (*p* = 0.02) and 0.63 (*p* = 0.005), respectively. These data suggest that IL-17 is a key pathological cytokine that potentially induces mitochondrial dysfunction in healthy bronchial fibroblasts and intensifies pre-existing mitochondrial damage in S-As fibroblasts.

Taking into consideration the effect of IL-17 on mitochondrial quantity and quality in bronchial fibroblasts, we next evaluated the significance of this change on cell fate. As seen in Figure 3D, flow cytometric analysis showed that IL-17 weakened Annexin V staining in both NHBF and DHBF, which suggested that IL-17 protected these fibroblasts from apoptosis. Western blot analysis further indicated that IL-17 treatment increased the expression of survivin, an anti-apoptotic protein, to a greater extent in DHBF than that in NHBF (Figure 3E). Taken together, these findings suggest that IL-17 mediated mitochondrial dysfunction may be associated with increased survival of bronchial fibroblasts.

### IL-17 Impairs Mitochondrial Quality Control in Bronchial Fibroblasts

**Figure 4:**
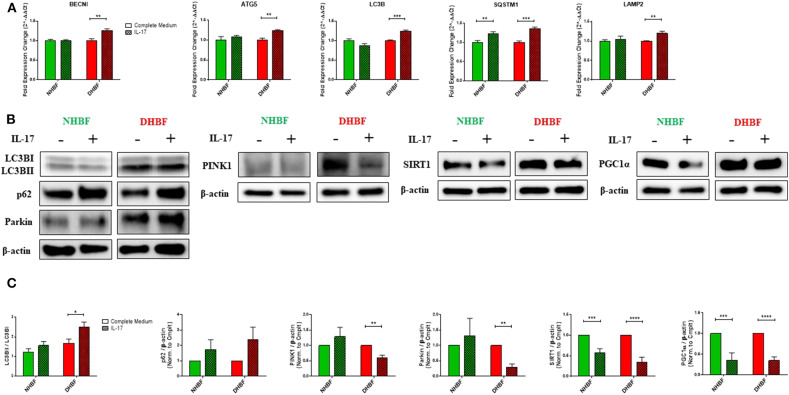
IL-17 impairs mitochondrial quality control in bronchial fibroblasts. NHBF and DHBF were serum-starved for 24 h and thereafter, cultured in DMEM complete medium without or with IL-17 (25 ng/ml) for 48 h for mRNA analysis and 96 h for protein analysis. **(A)** mRNA expression of autophagy genes, BECN1, ATG5, LC3B, SQSTM1, and LAMP2 in NHBF and DHBF upon IL-17 stimulation was analyzed by qPCR and expressed as fold expression change relative to the respective untreated control post normalization to housekeeping gene 18 s rRNA. Data representative of *n* = 3. **(B)** Representative immunoblots and **(C)** densitometric analysis of autophagy markers, with LC3B lipidation represented as the ratio of LC3BII to LC3BI and p62 expression, mitophagy markers, PINK1 and Parkin, and mitochondrial biogenesis markers, SIRT1, and PGC1α. Data representative of three independent experiments. β-actin was used as loading control. Data presented as mean ± SEM after normalization to the respective untreated control (Complete Medium). Statistical significance assessed by unpaired *t*-test with multiple comparisons using the Holm-Sidak method. *p < 0.05, **p < 0.01, ***p < 0.001, ****p < 0.0001.

We next studied the impact of IL-17 on autophagy in NHBF and DHBF by culturing the fibroblasts with or without IL-17 for a duration of 48 and 96 h for mRNA and protein analyses, respectively. We then measured the mRNA levels of autophagy markers, BECN1, ATG5, LC3B, SQSTM1, and LAMP2. IL-17 significantly increased the mRNA expression of these markers in DHBF but no change was detected in NHBF, except for SQSTM1 which showed a statistically significant increase in NHBF as well (Figure 4A). Western blot analysis showed that IL-17 treatment increased LC3B lipidation (LC3BII/LC3BI ratio) from 1.23 to 1.56 in NHBF and from 1.68 to 2.5 (*p* = 0.02) in DHBF (Figure 4C). IL-17 also showed an increased trend in the protein expression of p62 in NHBF and DHBF (Figures 4B,C). These findings suggest that IL-17 further upregulates autophagy by elevating the expression of autophagy-related genes in S-As fibroblasts.

Next, we aimed to determine the direct effects of IL-17 on mitochondrial QC in bronchial fibroblasts. In NHBF, there was a trend toward an increase in the expression of PINK1 and Parkin with IL-17 treatment (Figures 4B,C). However, this was not accompanied by a corresponding increase in SIRT1 and PGC1α expression. In contrast, a marked decline in SIRT1 and PGC1α expression was noted in NHBF (Figures 4B,C). IL-17 thus, contributed to increased mitophagy and lowered biogenesis in non-asthmatic fibroblasts. Surprisingly, IL-17 stimulation led to a significant reduction in PINK1 and Parkin expression (Figures 4B,C) together with a significant drop in SIRT1 and PGC1α levels in DHBF (Figures 4B,C). These results suggest that chronic exposure to IL-17 disrupts the balance between mitophagy and mitochondrial biogenesis leading to impairment in the mitochondrial quality control machinery in bronchial fibroblasts.

### Autophagy Triggers IL-17 Induced Mitochondrial Dysfunction

**Figure 5:**
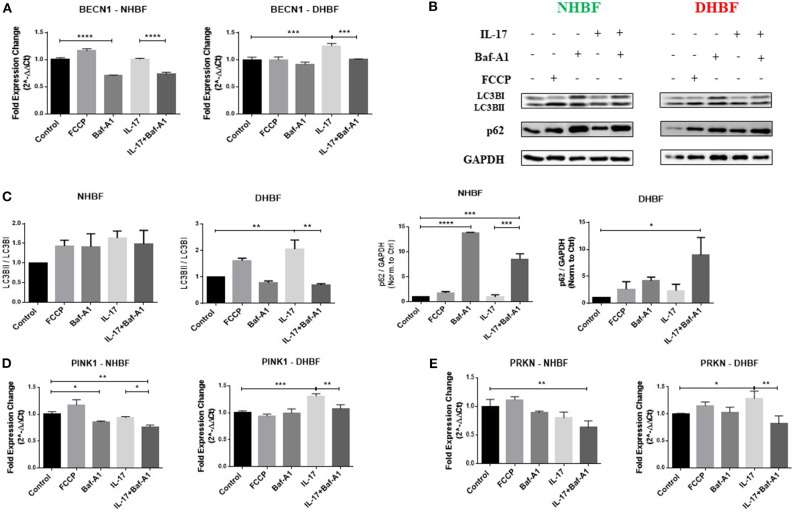
Autophagy triggers IL-17 induced mitochondrial dysfunction. NHBF and DHBF were serum-starved for 24 h, pre-treated with Bafilomycin-A1 (10 nM) for 4 h and thereafter, cultured in DMEM complete medium without or with IL-17 (25 ng/ml) for 48 h for mRNA analysis and 96 h for protein analysis. The cells were co-incubated with FCCP (10 μM) in the final 2 h of treatment. **(A)** mRNA expression of autophagy gene, BECN1, in NHBF (left panel) and DHBF (right panel) was analyzed by qPCR and expressed as fold expression change relative to the respective untreated control post normalization to housekeeping gene 18 s rRNA. Data representative of *n* = 3. **(B)** Representative immunoblots and **(C)** densitometric analysis depicting expression of autophagy markers, LC3B and p62, in NHBF and DHBF. GAPDH was used as loading control. Data representative of two independent experiments. **(D)** mRNA expression of PINK1 and **(E)** Parkin in NHBF (left panel) and DHBF (right panel) was analyzed by qPCR and expressed as fold expression change relative to the respective untreated control post normalization to housekeeping gene 18 s rRNA. Data representative of *n* = 3. Data presented as mean ± SEM after normalization to the respective untreated control. Statistical significance assessed by 1-way ANOVA with Tukey's multiple comparison tests. *p < 0.05, **p < 0.01, ***p < 0.001, ****p < 0.0001.

We observed that the enhanced autophagy levels in S-As fibroblasts was further elevated in response to IL-17. In order to study further the role of autophagy in IL-17 induced mitochondrial dysfunction, we pharmacologically inhibited autophagy in bronchial fibroblasts using bafilomycin-A1 (Baf-A1) that blocks autophagosomal fusion with lysosomes (27). Baf-A1, thus, causes the accumulation of autophagosomal vacuoles, which can be confirmed by the increased abundance of LC3BII and p62 in cells treated with Baf-A1. The bronchial fibroblasts were pre-treated with Baf-A1 at 10 nM for 4 h and then stimulated with IL-17 for up to 48 h for mRNA and up to 96 h for protein analyses. NHBF and DHBF were further co-incubated with 10 μM of FCCP in the final 2 h of treatment to induce mitochondrial uncoupling and to serve as a positive control for mitochondrial damage (28). FCCP treatment stimulated an increase in BECNI gene expression (Figure 5A) and increased accumulation of LC3BII and p62 proteins (Figures 5B,C) in NHBF, indicating the stimulation of autophagy machinery upon mitochondrial damage. Stimulation of PINK1 and PRKN gene expression was induced by FCCP in NHBF (Figures 5D,E), in agreement with the fact that FCCP induced mitochondrial uncoupling signals the removal of damaged mitochondria by increasing mitophagy. NHBF demonstrated a quick response to FCCP treatment within 2 h. Baf-A1 significantly decreased BECN1 gene expression (Figure 5A) and increased LC3B and p62 accumulation (Figures 5B,C) in NHBF, indicating successful inhibition of autophagy flux in NHBF. Baf-A1 treatment also suppressed mitophagy tagging in NHBF as a reduced trend in PINK1 and PRKN gene expression was observed (Figures 5D,E). Stimulation with IL-17 marginally increased the abundance of LC3BII and p62 in NHBF compared to time-matched untreated controls (Figures 5B,C). However, PINK1 and PRKN gene expression was not affected by IL-17 in NHBF (Figures 5D,E). Co-treatment with IL-17 and Baf-A1 reduced the PINK1 and PRKN gene expression to the lowest levels when compared to untreated controls (Figures 5D,E).

In DHBF, FCCP induced LC3BII and p62 accumulation (Figures 5B,C). However, PINK1 and PRKN gene expression was not affected by FCCP (Figures 5D,E), reinforcing the presence of depolarized mitochondria in DHBF. IL-17 induced activation of autophagy in DHBF as observed by elevated BECN1 gene expression (Figure 5A) and buildup of LC3BII and p62 proteins (Figures 5B,C) upon IL-17 treatment. A significant increase in PINK1 (1.3-fold) and PRKN (1.28-fold) gene expression (Figures 5D,E) by IL-17 was also noted in these fibroblasts, which suggested that IL-17 induced mitochondrial damage in DHBF signaling their detection and increased tagging by PINK1 and Parkin. Interestingly, co-treatment with IL-17 and Baf-A1 reversed the IL-17 mediated changes in PINK1 and PRKN mRNA levels (Figures 5D,E) in DHBF. Taken together, these findings suggest that IL-17 induced mitochondrial dysfunction in S-As fibroblasts is regulated by autophagy. Blocking autophagy reduced the expression of PINK1 and Parkin, indirectly suggesting an improvement in mitochondrial health resulting in a reduced demand for transcriptional regulation of PINK1 and Parkin-mediated mitophagy.

### Autophagy Triggers IL-17 Induced Pro-fibrotic Phenotype

**Figure 6:**
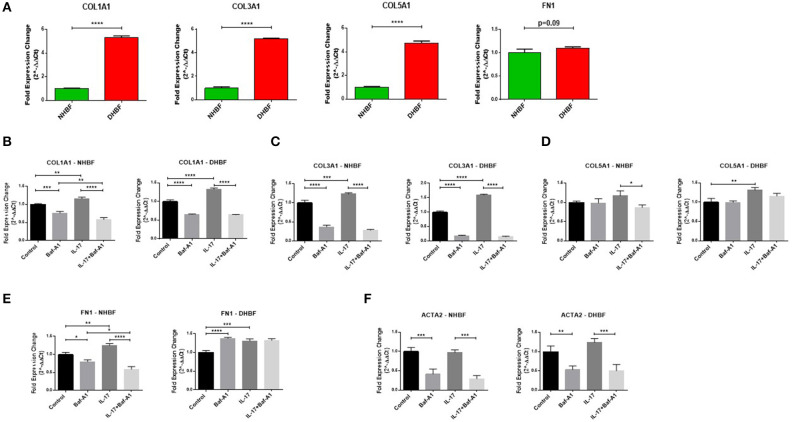
Autophagy triggers IL-17 induced pro-fibrotic phenotype. NHBF and DHBF were serum-starved for 24 h, pre-treated with Bafilomycin-A1 (10 nM) for 4 h and thereafter, cultured in DMEM complete medium without or with IL-17 (25 ng/ml) for 48 h. **(A)** mRNA expression of fibrotic genes, COL1A1, COL3A1, COL5A1 and FN1, at baseline in NHBF and DHBF. Data representative of *n* = 3. mRNA expression of **(B)** COL1A1, **(C)** COL3A1, **(D)** COL5A1, **(E)** FN1, and **(F)** ACTA2, in NHBF (left panel) and DHBF (right panel) was analyzed by qPCR and expressed as fold expression change relative to the untreated control post normalization to housekeeping gene 18s rRNA. Data representative of n=3. Data presented as mean ± SEM after normalization to NHBF or the respective untreated control. Statistical significance assessed by **(A)** unpaired two-tailed *t*-test or **(B–F)** 1-way ANOVA with Tukey's multiple comparison tests. *p < 0.05, **p < 0.01, ***p < 0.001, ****p < 0.0001.

Finally, we aimed to investigate whether there was an association between autophagy and IL-17 induced fibrogenesis in bronchial fibroblasts. Since asthmatic airways are characterized by increased deposition of ECM proteins, including collagen types I, III, and V (COL1A1, COL3A1, COL5A1), and fibronectin (FN1) (29), we first characterized their baseline expression in NHBF and DHBF. As expected, the S-As fibroblasts exhibited significantly higher mRNA expression of collagen subtypes, COL1A1 (*p* < 0.0001), COL3A1 (*p* < 0.0001), and COL5A1 (*p* < 0.0001) when compared to NHBF (Figure 6A). Although a trend toward an increase in mRNA expression of FN1 was noted in DHBF relative to NHBF, it was not statistically significant. Thus, the severe asthmatic fibroblasts used in this study demonstrated an increased pro-fibrotic profile when compared to their healthy counterparts.

We then evaluated whether the mRNA expression of these fibrotic genes as well as α-smooth muscle actin (ACTA2), a marker of myofibroblast differentiation, in bronchial fibroblasts was dependent on autophagy or IL-17 by inhibiting autophagy flux using Baf-A1. As shown in Figures 6B–F, at basal levels, blocking autophagy using Baf-A1 significantly decreased the expression of COL1A1, COL3A1, FN1 and ACTA2 in NHBF, and COL1A1, COL3A1, and ACTA2 in DHBF. IL-17 increased the transcriptional levels of COL1A1 (1.3-fold), COL3A1 (1.6-fold), COL5A1 (1.3-fold), and FN1 (1.2-fold) in DHBF. IL-17 also increased the gene expression of some of these ECM components in NHBF, but to a lower extent than in DHBF. Thus, IL-17 induced a potent fibrotic response in both non-asthmatic and S-As bronchial fibroblasts. Interestingly, co-treatment with Baf-A1 reversed the IL-17 induced increase in COL1A1, COL3A1, COL5A1 and FN1 in NHBF, and COL1A1, COL3A1, and ACTA2 in DHBF compared to their respective untreated controls. These findings suggest that IL-17 is a potent inducer of pro-fibrotic phenotype through induction of autophagy in bronchial fibroblasts.

## Discussion

Fibrosis is a challenging pathophysiological condition to treat in asthmatics. In fact, the inability of current asthmatic drugs to reverse fibrosis adds to the burden in asthmatic patients (30). In the present study, we investigated the effects of IL-17 on mitochondrial dysfunction and fibrosis in non-asthmatic and S-As fibroblasts. To our knowledge, this is the first study to demonstrate that IL-17 accelerated mitochondrial dysfunction and fibrosis in bronchial fibroblasts, but to a significantly greater extent in S-As fibroblasts when compared to non-asthmatic controls. This induction was further shown to be regulated by the activation of autophagy in these fibroblasts. Our data suggested that the pre-existing mitochondrial damage and fibrotic phenotype in S-As fibroblasts were further amplified by IL-17.

Mitochondrial QC machinery is essential for mitochondrial homeostasis and induction of mitochondrial damage activates this machinery to re-establish homeostasis. We first studied the mitochondrial QC mechanisms of mitophagy and mitochondrial biogenesis as an indicator of mitochondrial damage. Enhanced autophagy in S-As fibroblasts was associated with increased levels of mitophagy and biogenesis compared to healthy controls (Figures 1A,B). This suggested enhanced mitochondrial damage in S-As fibroblasts resulting in their continuous turnover. This was confirmed by the reduced mitochondrial membrane potential in these fibroblasts when compared to their healthy counterparts (Figure 1D). Additionally, the comparable mitochondrial mass between the two groups of fibroblasts (Figure 1C) reinforced the continuous turnover of damaged mitochondria.

PINK1 accumulation is a characteristic of mitochondrial damage and intense PINK1 immunoreactivity was observed in S-As airway biopsies (Figure 2B), which further confirmed increased mitochondrial damage in S-As airways than in healthy airways. The observed increase in autophagy and PINK1 immunoreactivity in S-As fibroblasts may be attributed to their prolonged exposure to increased levels of IL-17, which is known to be upregulated in the airway tissue microenvironment in severe asthma (6).

Because of the increasing implications of the pro-inflammatory and pro-fibrotic roles of IL-17 in severe asthma, we investigated the involvement of IL-17 in the mitochondrial dysfunction observed in S-As fibroblasts. The effects of IL-17 were more evident in S-As fibroblasts than in their healthy counterparts. In our study, we showed a 5-fold increase in IL-6 expression in NHBF and an 8-fold increase in DHBF upon IL-17 stimulation (Figure 3A). IL-6 is an important regulator of pathogenesis in asthma and has also been implicated in subepithelial fibrosis and airway remodeling in asthma (31). IL-6 is also vital in Th17 biology as it is essential for the differentiation of naïve T cells into Th17 cells, which are key producers of IL-17 (32). Furthermore, IL-6 stimulation of neutrophils from asthmatics significantly increased their production of IL-17A and IL-17F cytokines (33). Therefore, the induction of IL-6 expression in turn in structural bronchial fibroblasts by IL-17 creates a feedforward loop that sustains tissue inflammation by recruiting neutrophils and other immune cells to inflamed lung tissues thereby contributing to persistent airway tissue remodeling (34).

Furthermore, IL-17 significantly decreased the mitochondrial membrane potential and mitochondrial mass in S-As fibroblasts (Figures 3B,C). At the same time, a tendency toward a drop in ΔΨm and mitochondrial mass was noted in the healthy fibroblasts (Figures 3B,C). These hallmark features indicated that an IL-17 rich microenvironment renders the healthy fibroblasts vulnerable to mitochondrial malfunction and intensifies the pre-existing mitochondrial dysfunction in S-As fibroblasts. Taken together, our findings suggest that S-As fibroblasts show greater susceptibility to IL-17.

IL-17 induced reduction in mitochondrial mass implies enhanced mitochondrial degradation through mitophagy. As expected, IL-17 increased the expression of autophagy-related genes (Figure 4A), LC3B lipidation and protein levels of p62 (Figures 4B,C), to a statistically significant extent in S-As fibroblasts than in healthy. Interestingly, IL-17 treatment induced an increase in mitophagy in the healthy but it did not reach statistical significance, while a significant decline in mitophagy was observed in the S-As fibroblasts upon IL-17 stimulation (Figures 4B,C). The associated decline in mitochondrial biogenesis in both group of fibroblasts (Figures 4B,C) reflect an impairment in the mitochondrial QC machinery as a result of prolonged exposure to IL-17. This supports the notion that due to the innate phenotypic heterogeneity between non-asthmatic and S-As fibroblasts, they display unique responses to IL-17 stimulation.

Autophagy has emerged as a key player of cell survival in asthma (35). Increased LC3B expression exerted a cytoprotective role and inhibited hypoxia-induced epithelial apoptosis in lung epithelial cells (36). In addition to disabling the mitochondrial QC machinery, IL-17 inhibited apoptosis by decreasing Annexin V staining (Figure 3D) and inducing an increase in the expression of anti-apoptotic protein, Survivin (Figure 3E) in both non-asthmatic and S-As fibroblasts. IL-17 was previously reported to impair apoptosis in RA synovial fibroblasts through the activation of autophagy (16) and our findings are in accordance with this study.

We speculate that as a result of IL-17 induced mitochondrial damage, the healthy and S-As fibroblasts endure this damage by increasing their basal autophagy to pathological levels. In the presence of IL-17, the declining ΔΨm in the otherwise healthy mitochondria in healthy fibroblasts trigger an increase in mitophagy to mitigate the damage. However, the increased mitophagy is not paralleled by an increase in biogenesis. On the contrary, the continuous dissipation of ΔΨm caused by IL-17 in mitochondria with pre-existing mitochondrial abnormalities overwhelms the mitophagy and biogenesis machinery resulting in their malfunction in S-As fibroblasts. Nevertheless, the decreased mitochondrial mass in these fibroblasts may suggest the recruitment of alternative mitochondrial degradation mechanisms, including proteasomes, intramitochondrial proteolytic systems or vacuole/lysosome-mediated pathway (37), which may help prevent the accumulation of dysfunctional mitochondria and hence, overcome mitochondria-induced apoptosis (38). This may explain the IL-17 induced increased persistence of the healthy and S-As fibroblasts despite their mitochondrial dysfunction.

The observation of increased autophagy and mitochondrial dysfunction brought about by IL-17 in S-As fibroblasts led us to hypothesize that IL-17 induced autophagy regulates mitochondrial dysfunction as well as promotes the fibrotic phenotype of these diseased fibroblasts. Accordingly, inhibition of autophagy using Baf-A1 reversed the IL-17 mediated increase in PINK1 and PRKN gene expression in S-As fibroblasts (Figures 5D,E). Co-incubation with IL-17 and Baf-A1 also brought about a significant reduction in PINK1 and PRKN gene expression in the healthy fibroblasts when compared to their untreated controls (Figures 5D,E). Since increased PINK1 and PRKN gene expression is induced upon mitochondrial damage, their decreased gene expression may imply a reduction in mitochondrial dysfunction.

It was however interesting to note that the IL-17 induction of PINK1 and PRKN gene expression in S-As fibroblasts (Figures 5D,E) did not correlate with the expression of their protein products (Figures 4B,C). Since IL-17 induced mitochondrial dysfunction in these fibroblasts, the transcriptional upregulation of PINK1 and PRKN represents a consequential response. However, it is plausible that IL-17 may also exert an influence on the post-transcriptional factors, including protein translation, post-translational events and protein degradation, thereby, impairing the mitochondrial QC machinery within these fibroblasts. To this regard, a study by Chowdhury et al. showed the ability of IL-17A to interact with microRNAs and thereby affect AUBps protein binding to mRNA facilitating either mRNA decay or stabilization (39). At the same time, the kinetics of IL-17 induced gene and protein expression is currently not well-understood and future studies using pulse-chase labeling may provide an improved understanding of this mechanism.

We, along with many others, have previously reported that subepithelial fibrosis in asthmatic airways is characterized by increased deposition of collagens, specifically collagen types I, III and V, and fibronectin (29, 40–42) as well as increased myofibroblast differentiation (43). Accordingly, the S-As fibroblasts demonstrated significantly enhanced gene expression of these collagen subtypes when compared to their healthy counterparts (Figure 6A). More importantly, we have also showed the association between increased levels of IL-17 and collagen types I and III in severe asthmatic bronchial tissues (42). In a study of orbital fibroblasts in thyroid-associated ophthalmopathy, IL-17 promoted the gene expression of collagen types I and III, and ACTA2 (44). In line with these previous studies, IL-17 augmented the gene expression of COL1A1, COL3A1, COL5A1, and FNI to a greater extent in S-As fibroblasts than their healthy counterparts (Figures 6B–E). Interestingly, IL-17 did not induce myofibroblast differentiation in bronchial fibroblasts (Figure 6F). This could perhaps be because parenchymal fibroblasts being major producers of α-smooth muscle actin (α-SMA) contributed largely to the myofibroblastic phenotype as opposed to bronchial fibroblasts (45). Although IL-17 has previously been reported to enhance the expression of pro-fibrotic genes, its ability to induce ACTA2 expression was found to vary from cell to cell. For instance, IL-17 alone could not induce ACTA2 expression in primary human hepatic stellate cells (46). On the other hand, IL-17A promoted ACTA2 expression in orbital fibroblasts in thyroid-associated ophthalmopathy (44). Alternatively, autophagy was found to regulate myofibroblast differentiation in bronchial fibroblasts.

A recent study demonstrated selective activation of autophagy in a cell context-dependent manner in asthma (47). In this study, autophagy was found to be critical in the development of airway remodeling with multiple autophagy markers showing positive staining in tissue sections of asthmatic airways. Moreover, inhibiting autophagy using chloroquine was found to attenuate airway inflammation, airway hyperresponsiveness and airway remodeling, including a reduction in α-SMA immunoreactivity in the airways, in allergic asthmatic mice. We have also previously reported that dysregulation of autophagy is associated with subepithelial fibrosis in the airways of refractory asthmatics (19). In this study, ATG5 gene expression positively correlated with COL5A1 expression in bronchial biopsies from refractory asthmatics. Interestingly, our results are in line with these earlier studies as we show that co-incubation of bronchial fibroblasts with Baf-A1 significantly reduced COL1A1, COL3A1, FN1, and ACTA2 gene expression (Figures 6B–F). Additionally, inhibition of autophagy also blocked the IL-17 mediated increase in pro-fibrotic gene signature in both groups of fibroblasts.

## Conclusions

In summary, our data suggest that IL-17 induces mitochondrial dysfunction and pro-fibrotic signature through the activation of autophagy in bronchial fibroblasts. This provides insight into a potential pathway that contributes to fibrosis in severe asthmatic airways, and reveal the therapeutic potential of targeting autophagy to subdue fibrosis, particularly in severe asthmatic individuals.

## Data Availability Statement

The data generated and/or analyzed during the current study are available from the corresponding author on reasonable request.

## Ethics Statement

The primary bronchial fibroblasts, and paraffin slides from non-asthmatic controls used in our study were obtained from the Biobank at the Quebec Respiratory Health Research Network. The original study was approved by institutional review board (MUHC REB number BMB-02-039-t) and the subjects had provided written informed consent.

The severe asthmatic bronchial biopsies were obtained from patients recruited at the Severe Asthma Clinic in the Pulmonary Medicine department at Rashid Hospital. The study was approved by the Dubai Health Authority and Dubai Scientific Research Ethics Committee (DSREC-11/2017_04). The patients received a detailed description of the study from the nurse and researchers, and samples were collected after their written informed consent.

## Author Contributions

QH, RR, KB, and RHal: conceptualization. RR, AA, SR, LS, and MH: data curation. RR, AA, and IH: formal analysis. QH, BM, and RHam: funding acquisition and project administration. RR, KB, and RHal: investigation. RR: methodology and validation. QH, RHam, and RO: resources. QH, RHal, BM, and RHam: supervision. RR, AA, and IH: visualization. RR and SA: writing – original draft. RR, SA, KB, and RHal: writing – review and editing.

## Conflict of Interest

The authors declare that the research was conducted in the absence of any commercial or financial relationships that could be construed as a potential conflict of interest.

## Abbreviations

ECM: Extracellular matrix
NHBF: Normal human bronchial fibroblasts
DHBF: Diseased human bronchial fibroblasts
COPD: Chronic obstructive pulmonary disease
IPF: Idiopathic pulmonary fibrosis
S-As: Severe asthmatic
DMEM: Dulbecco's Modified Eagle's Medium
FBS: Fetal bovine serum
FCCP: Carbonyl cyanide-4-(trifluoromethoxy) phenylhydrazone
qPCR: Quantitative Polymerase Chain Reaction
QC: Quality control
LC3B: Microtubule-associated protein 1 light chain 3B
LAMP2: Lysosomal-associated membrane protein 2
PINK1: PTEN-induced putative kinase 1
PRKN: Parkin
SIRT1: Sirtuin 1
PGC1α: Peroxisome proliferator-activated receptor gamma coactivator 1 alpha
ΔΨm: Mitochondrial membrane potential
BECN1: Beclin 1
ATG5: Autophagy related 5
SQSTM1/p62: Sequestosome-1
Baf-A1: Bafilomycin-A1
COL1A1: Collagen, type I, alpha 1
COL3A1: Collagen, type III, alpha 1
COL5A1: Collagen, type V, alpha 1
FN1: Fibronectin.

## References

[1] Kis K Liu X Hagood JS. Myofibroblast differentiation and survival in fibrotic disease. Expert Rev Mol Med. (2011) 13:e27. 10.1017/S1462399411001967 21861939 PMC5675569

[2] Benayoun L Druilhe A Dombret MC Aubier M Pretolani M. Airway structural alterations selectively associated with severe asthma. Am J Respir Crit Care Med. (2003)167:1360–8. 10.1164/rccm.200209-1030OC 12531777

[3] Weitoft M Andersson C Andersson-Sjoland A Tufvesson E Bjermer L Erjefalt J. Controlled and uncontrolled asthma display distinct alveolar tissue matrix compositions. Respir Res. (2014) 15:67. 10.1186/1465-9921-15-67 24950767 PMC4089934

[4] Boser SR Mauad T Araujo-Paulino BB Mitchell I Shrestha G Chiu A. Myofibroblasts are increased in the lung parenchyma in asthma. PLoS ONE. (2017) 12:e0182378. 10.1371/journal.pone.0182378 28787016 PMC5546673

[5] Ramakrishnan RK Al Heialy S Hamid Q. Role of IL-17 in asthma pathogenesis and its implications for the clinic. Expert Rev Respir Med. (2019) 13:1057–68. 10.1080/17476348.2019.1666002 31498708

[6] Al-Ramli W Prefontaine D Chouiali F Martin JG Olivenstein R Lemiere C. T(H)17-associated cytokines (IL-17A and IL-17F) in severe asthma. J Allergy Clin Immunol. (2009) 123:1185–7. 10.1016/j.jaci.2009.02.024 19361847

[7] Shannon J Ernst P Yamauchi Y Olivenstein R Lemiere C Foley S. Differences in airway cytokine profile in severe asthma compared to moderate asthma. Chest. (2008) 133:420–6. 10.1378/chest.07-1881 18071017

[8] Woodruff PG Modrek B Choy DF Jia G Abbas AR Ellwanger A. T-helper type 2-driven inflammation defines major subphenotypes of asthma. Am J Respir Crit Care Med. (2009) 180:388–95. 10.1164/rccm.200903-0392OC 19483109 PMC2742757

[9] Molet S Hamid Q Davoine F Nutku E Taha R Page N. IL-17 is increased in asthmatic airways and induces human bronchial fibroblasts to produce cytokines. J Allergy Clin Immunol. (2001) 108:430–8. 10.1067/mai.2001.117929 11544464

[10] Loubaki L Hadj-Salem I Fakhfakh R Jacques E Plante S Boisvert M. Co-culture of human bronchial fibroblasts and CD4+ T cells increases Th17 cytokine signature. PLoS ONE. (2013) 8:e81983. 10.1371/journal.pone.0081983 24349168 PMC3857794

[11] Camargo LDN Righetti RF Aristoteles L Dos Santos TM de Souza FCR Fukuzaki S. Effects of Anti-IL-17 on inflammation, remodeling, and oxidative stress in an experimental model of asthma exacerbated by LPS. Front Immunol. (2017) 8:1835. 10.3389/fimmu.2017.01835 29379497 PMC5760512

[12] Heinzmann A Thoma C Dietrich H Deichmann KA. Identification of common polymorphisms in the mitochondrial genome. Allergy. (2003) 58:830–1. 10.1034/j.1398-9995.2003.00223.x 12859577

[13] Mabalirajan U Dinda AK Kumar S Roshan R Gupta P Sharma SK. Mitochondrial structural changes and dysfunction are associated with experimental allergic asthma. J Immunol. (2008) 181:3540–8. 10.4049/jimmunol.181.5.3540 18714027

[14] Aguilera-Aguirre L Bacsi A Saavedra-Molina A Kurosky A Sur S Boldogh I. Mitochondrial dysfunction increases allergic airway inflammation. J Immunol. (2009) 183:5379–87. 10.4049/jimmunol.0900228 19786549 PMC3028535

[15] Servais S Boussouar A Molnar A Douki T Pequignot JM Favier R. Age-related sensitivity to lung oxidative stress during ozone exposure. Free Radical Res. (2005) 39:305–16. 10.1080/10715760400011098 15788235

[16] Kim EK Kwon JE Lee SY Lee EJ Kim DS Moon SJ. IL-17-mediated mitochondrial dysfunction impairs apoptosis in rheumatoid arthritis synovial fibroblasts through activation of autophagy. Cell Death Dis. (2017) 8:e2565. 10.1038/cddis.2016.490 28102843 PMC5386390

[17] Zhou J An X Dong J Wang Y Zhong H Duan L. IL-17 induces cellular stress microenvironment of melanocytes to promote autophagic cell apoptosis in vitiligo. FASEB J. (2018) 32:4899–916. 10.1096/fj.201701242RR 29613836

[18] Panariti A Baglole CJ Sanchez V Eidelman DH Hussain S Olivenstein R. Interleukin-17A and vascular remodelling in severe asthma; lack of evidence for a direct role. Clin Exp Allergy. (2018) 48:365–78. 10.1111/cea.13093 29337379

[19] Poon AH Choy DF Chouiali F Ramakrishnan RK Mahboub B Audusseau S. Increased autophagy-related 5 gene expression is associated with collagen expression in the airways of refractory asthmatics. Front Immunol. (2017) 8:355. 10.3389/fimmu.2017.00355 28424691 PMC5372794

[20] Ichikawa T Panariti A Audusseau S Mogas AK Olivenstein R Chakir J. Effect of bronchial thermoplasty on structural changes and inflammatory mediators in the airways of subjects with severe asthma. Respir Med. (2019) 150:165–72. 10.1016/j.rmed.2019.03.005 30961946

[21] Tanaka Y Guhde G Suter A Eskelinen EL Hartmann D Lullmann-Rauch R. Accumulation of autophagic vacuoles and cardiomyopathy in LAMP-2-deficient mice. Nature. (2000) 406:902–6. 10.1038/35022595 10972293

[22] Xiao B Deng X Zhou W Tan EK. Flow cytometry-based assessment of mitophagy using MitoTracker. Front Cell Neurosci. (2016) 10:76. 10.3389/fncel.2016.00076 27065339 PMC4811937

[23] Wang C Youle RJ. The role of mitochondria in apoptosis^*^. Ann Rev Gene. (2009) 43:95–118. 10.1146/annurev-genet-102108-134850 19659442 PMC4762029

[24] Marino G Niso-Santano M Baehrecke EH Kroemer G. Self-consumption: the interplay of autophagy and apoptosis. Nat Rev Mol Cell Biol. (2014) 15:81–94. 10.1038/nrm3735 24401948 PMC3970201

[25] Pickrell AM Youle RJ. The roles of PINK1, parkin, and mitochondrial fidelity in Parkinson's disease. Neuron. (2015) 85:257–73. 10.1016/j.neuron.2014.12.007 25611507 PMC4764997

[26] Dessalle K Narayanan V Kyoh S Mogas A Halayko AJ Nair P. Human bronchial and parenchymal fibroblasts display differences in basal inflammatory phenotype and response to IL-17A. Clin Exp Allergy. (2016) 46:945–56. 10.1111/cea.12744 27079765

[27] Ghavami S Mutawe MM Schaafsma D Yeganeh B Unruh H Klonisch T. Geranylgeranyl transferase 1 modulates autophagy and apoptosis in human airway smooth muscle. Am J Physiol Lung Cell Mol Physiol. (2012) 302:L420–8. 10.1152/ajplung.00312.2011 22160308

[28] Berezhnov AV Soutar MP Fedotova EI Frolova MS Plun-Favreau H Zinchenko VP. Intracellular pH modulates autophagy and mitophagy. J Biol Chem. (2016) 291:8701–8. 10.1074/jbc.M115.691774 26893374 PMC4861439

[29] Wilson JW Li X. The measurement of reticular basement membrane and submucosal collagen in the asthmatic airway. Clin Exp Allergy. (1997) 27:363–71. 10.1046/j.1365-2222.1997.600864.x 9146928

[30] Durrani SR Viswanathan RK Busse WW. What effect does asthma treatment have on airway remodeling? Current perspectives. J Allergy Clin Immunol. (2011) 128:439–48; quiz 49–50. 10.1016/j.jaci.2011.06.002 21752441

[31] Rincon M Irvin CG. Role of IL-6 in asthma and other inflammatory pulmonary diseases. Int J Biol Sci. (2012) 8:1281–90. 10.7150/ijbs.4874 23136556 PMC3491451

[32] Zhou L Ivanov II Spolski R Min R Shenderov K Egawa T. IL-6 programs T(H)-17 cell differentiation by promoting sequential engagement of the IL-21 and IL-23 pathways. Nat Immunol. (2007) 8:967–74. 10.1038/ni1488 17581537

[33] Halwani R Sultana A Vazquez-Tello A Jamhawi A Al-Masri AA Al-Muhsen S. Th-17 regulatory cytokines IL-21, IL-23, and IL-6 enhance neutrophil production of IL-17 cytokines during asthma. J Asthma. (2017) 54:893–904. 10.1080/02770903.2017.1283696 28635548

[34] Iwakura Y Ishigame H Saijo S Nakae S. Functional specialization of interleukin-17 family members. Immunity. (2011) 34:149–62. 10.1016/j.immuni.2011.02.012 21349428

[35] Jyothula SS Eissa NT. Autophagy and role in asthma. Curr Opin Pulm Med. (2013) 19:30–5. 10.1097/MCP.0b013e32835b1150 23143196

[36] Tanaka A Jin Y Lee SJ Zhang M Kim HP Stolz DB. Hyperoxia-induced LC3B interacts with the Fas apoptotic pathway in epithelial cell death. Am J Respir Cell Mol Biol. (2012) 46:507–14. 10.1165/rcmb.2009-0415OC 22095627 PMC3359946

[37] Mijaljica D Prescott M Devenish RJ. Different fates of mitochondria: alternative ways for degradation? Autophagy. (2007) 3:4–9. 10.4161/auto.3011 16929167

[38] Kubli DA Gustafsson AB. Mitochondria and mitophagy: the yin and yang of cell death control. Circ Res. (2012) 111:1208–21. 10.1161/CIRCRESAHA.112.265819 23065344 PMC3538875

[39] Chowdhury S Dijkhuis A Steiert S Lutter R. IL-17 attenuates degradation of ARE-mRNAs by changing the cooperation between AU-binding proteins and microRNA16. PLoS Gene. (2013) 9:e1003747. 10.1371/journal.pgen.1003747 24086143 PMC3784493

[40] Ignotz RA Massague J. Transforming growth factor-beta stimulates the expression of fibronectin and collagen and their incorporation into the extracellular matrix. J Biol Chem. (1986) 261:4337–45. 3456347

[41] Hoshino M Nakamura Y Sim J Shimojo J Isogai S. Bronchial subepithelial fibrosis and expression of matrix metalloproteinase-9 in asthmatic airway inflammation. J Allergy Clin Immunol. (1998) 102:783–8. 10.1016/S0091-6749(98)70018-1 9819295

[42] Chakir J Shannon J Molet S Fukakusa M Elias J Laviolette M. Airway remodeling-associated mediators in moderate to severe asthma: effect of steroids on TGF-beta, IL-11, IL-17, and type I and type III collagen expression. J Allergy Clin Immunol. (2003) 111:1293–8. 10.1067/mai.2003.1557 12789232

[43] Brewster CE Howarth PH Djukanovic R Wilson J Holgate ST Roche WR. Myofibroblasts and subepithelial fibrosis in bronchial asthma. Am J Respir Cell Mol Biol. (1990) 3:507–11. 10.1165/ajrcmb/3.5.507 2223105

[44] Fang S Huang Y Wang S Zhang Y Luo X Liu L. IL-17A Exacerbates fibrosis by promoting the proinflammatory and profibrotic function of orbital fibroblasts in TAO. J Clin Endocrinol Metabol. (2016) 101:2955–65. 10.1210/jc.2016-1882 27224264

[45] Pechkovsky DV Hackett TL An SS Shaheen F Murray LA Knight DA Human lung parenchyma but not proximal bronchi produces fibroblasts with enhanced TGF-beta signaling and alpha-SMA expression. Am J Respir Cell Mol Biol. (2010) 43:641–51. 10.1165/rcmb.2009-0318OC 20061511

[46] Fabre T Kared H Friedman SL Shoukry NH. IL-17A enhances the expression of profibrotic genes through upregulation of the TGF-beta receptor on hepatic stellate cells in a JNK-dependent manner. J Immunol. (2014) 193:3925–33. 10.4049/jimmunol.1400861 25210118 PMC4185218

[47] McAlinden KD Deshpande DA Ghavami S Xenaki D Sohal SS Oliver BG. Autophagy activation in asthma airways remodeling. Am J Respir Cell Mol Biol. (2019) 60:541–53. 10.1165/rcmb.2018-0169OC 30383396 PMC6503620

